# Racial Disparities in Outcomes Following Open Treatment of Pediatric Femoral Shaft Fractures

**DOI:** 10.7759/cureus.33149

**Published:** 2022-12-30

**Authors:** Alisa Malyavko, Theodore Quan, Denver Kraft, Abhay Mathur, Pradip Ramamurti, Sean Tabaie

**Affiliations:** 1 Orthopaedic Surgery, George Washington University School of Medicine and Health Sciences, Washington DC, USA; 2 Orthopaedic Surgery, Georgetown University School of Medicine, Washington DC, USA; 3 Orthopaedic Surgery, University of Virginia School of Medicine, Charlottesville, USA; 4 Orthopaedic Surgery, Children's National Hospital, Washington DC, USA

**Keywords:** minority groups, complications, underrepresented patients, race, open treatment, femoral shaft fractures

## Abstract

Introduction

Femoral shaft fractures are a common pediatric injury that can require non-operative or operative management. Several studies have shown that race impacts pain management and a number of emergency department visits in the pediatric femur fracture population. This study aimed to investigate any association between pediatric patient race and number of comorbidities, 30-day postoperative outcomes, and length of stay following open surgical treatment of femoral shaft fractures.

Methods

Pediatric patients who underwent open treatment of femoral shaft fracture were identified in the National Surgical Quality Improvement Program-Pediatric database from 2012-2019. Patients were categorized into two cohorts: White and underrepresented minority (URM). URM groups included Black or African American, Hispanic, Native American or Alaskan, and Native Hawaiian or Pacific Islander. Demographics, comorbidities, and postoperative complications were compared using bivariate and multivariable regression analyses.

Results

Of the 5,284 pediatric patients who underwent open treatment of femoral shaft fracture, 3,650 (69.1%) were White, and 1,634 (30.9%) were URM. Compared to White patients, URM patients were more likely to have a higher American Society of Anesthesiologists score (p=0.012), more likely to have pulmonary comorbidities (p=0.005), require preoperative blood transfusion (p=0.006), and have an increased risk of prolonged hospital stay (OR 2.36; p=0.007).

Conclusion

Pediatric URM patients undergoing open treatment of femoral shaft fractures have an increased risk of extended hospital stay postoperatively compared to White patients. As the racial and ethnic constitution of the pediatric population changes, understanding racial and ethnic health disparities will be crucial to providing equitable care to all patients.

## Introduction

Femoral shaft fractures are the most common pediatric lower extremity fracture and the leading cause among pediatric orthopedic injuries that require hospitalization [1,2]. The incidence of pediatric femoral shaft fractures in the United States is estimated to be 20 per 100,000 children per year [1, 3-6]. The mechanisms of injury leading to a femoral shaft fracture vary based on the patient's age. Falls are the most common mechanism for children under the age of six, while high-impact sports, motor vehicle-pedestrian accidents, and motor vehicle accidents are more common among preadolescents and adolescents [1-3]. Hunter et al. found that black males between the ages of 14 and 17 years were at the highest risk for femoral shaft fractures. Additionally, black children across all age groups had an increased risk of motor vehicle-pedestrian accidents compared to White children [3].

Many studies have evaluated various treatment modalities for pediatric femoral shaft fractures and have created evidence-based treatment algorithms [2, 7-9]. Management depends on the patient's age, weight, fracture pattern, mobility, possible complications, and surgeon expertise [2,7]. For patients over the age of five years, operative management with flexible or rigid intramedullary nails or submuscular plates allows for a more timely return to prior levels of mobility and function [2,8].

Recently, increased literature regarding healthcare disparities has been published in the adult population, with more limited attention on the pediatric population [10-14]. A study by Patel et al. investigated the healing pattern of osteochondritis dissecans lesions in pediatric knees. They found that black patients had 6.7 times greater odds of unsuccessful healing when compared to the White patient cohort [15]. Another study by Kulaylat et al. looked at factors impacting the transfer of pediatric trauma patients from adult trauma centers to pediatric trauma centers and found that there was an increased rate of interfacility transfer for uninsured pediatric patients and non-White patients [16].

The aim of this study was to determine whether an association exists between race and 30-day outcomes after surgical treatment of femoral shaft fractures in pediatric patients. We hypothesized that there would be differences in preoperative comorbidities and postoperative complications between the White and underrepresented minority (URM) groups. Specifically, we hypothesized that pediatric patients from URM groups would have an increased number of comorbidities, more postoperative complications, an increased operative time, and an extended length of hospital stay.

## Materials and methods

This retrospective cohort study used the American College of Surgeons National Surgical Quality Improvement Program-Pediatric (ACS NSQIP-P) database, a national database that had been shown to have a high inter-observer agreement rate [17,18]. Trained reviewers at each participating site collect demographic, comorbidity, postoperative, and other variables [19]. In this database, patient information is de-identified and can be used to follow the clinical course of each individual patient [20-22].

Patient selection

To identify all patients undergoing open treatment of femoral shaft fracture from 2012-2019, current procedural terminology (CPT) codes 27506 and 27507 were used. Patients younger than 18 years old were included in the study. Patients who were missing key demographic information were excluded. Two patient groups were defined: patients who were White and patients from URM groups. Patients from URM groups included those who were black or African American, Hispanic, Native American or Alaskan, and Native Hawaiian or Pacific Islander, consistent with the existing literature [23].

Baseline characteristics

Demographics and clinical characteristics were collected from the database, including age, gender, American Society of Anesthesiologists (ASA) score, and comorbidities. Comorbidities included pulmonary (a history of asthma, oxygen support, structural airway abnormalities, tracheostomy, chronic lung disease, or ventilator dependence), cardiac (any cardiac risk factors, previous cardiac surgery, cardiopulmonary resuscitation within seven days prior to surgery, or inotropic support at the time of surgery), neurological (seizure disorder, developmental delay, structural central nervous system abnormality, cerebral palsy, or neuromuscular disorder), gastrointestinal (esophageal, gastric, or intestinal disease), biliary (biliary, liver, or pancreatic disease). Immune disease, failure to thrive, nutritional support, bleeding or hematologic disorder, and preoperative blood transfusion were also recorded.

Postoperative complications

Postoperative complications within 30 days of surgery were assessed, including superficial or deep surgical site infections, superficial or deep wound dehiscence, pneumonia, urinary tract infection, seizure, cardiac arrest, bleeding requiring transfusion, venous thromboembolism, *Clostridium difficile* infection, sepsis, prolonged operation time, prolonged length of hospital stay, return to the operating room, readmission, and mortality. Extended operation time was defined as more than 155 minutes, which was one standard deviation above the mean operation time for the patients in this study. Extended length of stay was defined as more than 5.5 days, which is one standard deviation above the mean length of stay.

Statistical analysis

Demographics, comorbidities, and complications were compared between the two groups using Pearson's chi-squared test and analysis of variance. Postoperative complications were also compared between the cohorts with chi-squared tests. All demographic and comorbidity variables with a p-value <0.20 on bivariate analyses were included in a multivariable regression analysis to adjust for confounding variables [18,23]. Odds ratios and 95% confidence intervals were reported from the regression analysis results. Statistical significance was maintained at a p-value <0.05, and all analyses were done with the use of SPSS version 28 (IBM Inc., Armonk, New York) software.

## Results

Demographics and comorbidities

In total, 5,284 pediatric patients underwent open treatment of a femoral shaft fracture and were included in the analysis. Of these, 3,650 patients (69.1%) were White, and 1,634 (30.9%) were from URM groups. URM patients were more likely to have a higher ASA classification (p=0.012) (Table 1), to have more pulmonary comorbidities (p=0.005), and to require preoperative blood transfusion (p=0.006) (Table 2).

**Table 1:** Baseline characteristics among patients undergoing open treatment of femoral shaft fracture ¶Pearson's chi-squared test **Analysis of variance Bolding equals significance p<0.05 ASA - American Society of Anesthesiologists; SD - standard deviation

Demographics	White	Underrepresented minority	p-value
Total patients, n	3,650	1,634	
Sex, n (%)			0.052^¶^
Female	930 (25.5)	458 (28.0)	
Male	2,720 (74.5)	1,176 (72.0)	
ASA, n (%)			0.012^¶^
I	1,545 (52.0)	595 (46.9)	
II	1,068 (35.9)	514 (40.5)	
III	348 (11.7)	153 (12.0)	
IV	11 (0.4)	8 (0.6)	
Mean age, yrs (SD)	10.09 (3.90)	10.06 (3.96)	0.774**

**Table 2:** Comorbidities among patients undergoing open treatment of femoral shaft fracture ¶Pearson's chi-squared test Bolding equals significance p<0.05

Comorbidities	White	Underrepresented minority	p-value^ ¶^
Total patients, n	3,650	1,634	
Pulmonary comorbidity, n (%)	231 (6.3)	138 (8.4)	0.005
Cardiac comorbidity, n (%)	91 (2.5)	27 (1.7)	0.056
Neurological comorbidity, n (%)	330 (9.0)	162 (9.9)	0.313
Gastrointestinal comorbidity, n (%)	125 (3.4)	42 (2.6)	0.101
Biliary comorbidity, n (%)	5 (0.5)	0 (0.0)	0.174
Immune disease, n (%)	3 (0.3)	0 (0.0)	0.292
Failure to thrive, n (%)	4 (0.5)	1 (0.4)	0.740
Nutritional support, n (%)	82 (2.2)	44 (2.7)	0.326
Bleeding disorder, n (%)	3 (0.3)	1 (0.3)	0.930
Hematologic disorder, n (%)	44 (1.2)	25 (1.5)	0.337
Preoperative blood transfusion, n (%)	6 (0.2)	10 (0.6)	0.006

Complications

On bivariate analysis, patients who were from the URM cohort were more likely to require postoperative blood transfusion (p=0.009), have a prolonged operation time (p=0.007), and have an extended length of hospital stay (p=0.012) (Table 3). Following adjustment on multivariable regression analysis to control for potential confounders, pediatric patients from the URM cohort are at an increased risk of prolonged hospital stay (OR 2.356; 95% CI 1.270 to 4.371; p=0.007) when compared to patients who were white (Table 4).

**Table 3:** Bivariate analysis of postoperative complications of patients following femoral shaft fracture treatment ¶Pearson's chi-squared test Bolding equals significance p<0.05

Complications	White	Underrepresented minority	p-value^¶^
Total patients, n	3,650	1,634	
Superficial surgical site infection, n (%)	12 (0.3)	4 (0.2)	0.608
Deep surgical site infection, n (%)	2 (0.1)	1 (0.1)	0.928
Superficial wound dehiscence, n (%)	17 (0.8)	5 (0.5)	0.458
Deep wound dehiscence, n (%)	4 (0.1)	3 (0.2)	0.494
Pneumonia, n (%)	4 (0.1)	2 (0.1)	0.898
Urinary tract infection, n (%)	11 (0.3)	3 (0.2)	0.441
Seizure, n (%)	1 (0.0)	1 (0.1)	0.559
Cardiac arrest, n (%)	0 (0.0)	1 (0.1)	0.135
Postoperative transfusion, n (%)	73 (2.0)	52 (3.2)	0.009
Venous thromboembolism, n (%)	5 (0.1)	2 (0.1)	0.893
Clostridium difficile infection, n (%)	0 (0.0)	1 (0.1)	0.147
Sepsis, n (%)	2 (0.1)	0 (0.0)	0.344
Extended operation time (> 155 minutes), n (%)	415 (11.4)	229 (14.0)	0.007
Extended length of stay (> 5.5 days), n (%)	130 (3.6)	82 (5.0)	0.012
Reoperation, n (%)	74 (3.1)	27 (2.7)	0.513
Readmission, n (%)	79 (3.3)	35 (3.5)	0.823
Death, n (%)	0 (0.0)	1 (0.1)	0.135

**Table 4:** Multivariable regression analysis of postoperative complications of patients following femoral shaft fracture treatment Bolding equals significance p<0.05 CI - confidence interval

Complications	Underrepresented minority	
	P-value	Odds ratio (underrepresented minority/white) (95% CI)
Postoperative transfusion	0.147	1.843 (0.807 to 4.209)
Extended operation time (>155 minutes)	0.439	1.166 (0.790 to 1.720)
Extended length of stay (>5.5 days)	0.007	2.356 (1.270 to 4.371)

## Discussion

The pediatric population of the United States is ethnically and racially diverse. According to US Census Bureau data, in 2020, 50% of US children were White, 26% were Hispanic, 14% were black, 5% were Asian, and 5% were other non-Hispanic races. These percentages are predicted to change by 2050 to reflect an increasing percentage of children who are Hispanic, Black, Asian, and other non-Hispanic races [24]. Investigating and addressing racial and ethnic health disparities is crucial to continue providing the highest quality of pediatric care to all patients [25]. This analysis of the association between race and outcomes following open treatment of femoral shaft fractures found that pediatric patients from the URM cohort had an increased length of hospital stay but did not have any significant differences in 30-day postoperative complications when compared to patients who were White. Patients from the URM cohort did have an increased number of comorbidities but did not have an increased operative time.

Pediatric bone health is influenced by many factors, including nutrition, obesity, and systemic health conditions that can contribute to fracture rate and the need for treatment among pediatric patients [26]. Our study found that patients from URM groups were more likely to have higher ASA scores and pulmonary comorbidities. The difference in pulmonary comorbidities and ASA scores between each group may suggest a common risk factor for URM patients. Patients in the URM group also had an increased rate of postoperative blood transfusion, but evaluating the nature of the injury or cause of this transfusion requirement was outside the scope of this study. Prior studies have found that preoperative blood transfusions are associated with increased postoperative complications, including infection, postoperative blood transfusion, reintubation, and reoperation. Additionally, both preoperative and perioperative blood transfusions have been associated with an extended length of hospital stay [27,28]. While our study did not find any significant difference in postoperative complications between the cohorts, the URM cohort did have a significantly longer hospital length of stay.

Although our study did not show significant differences in 30-day postoperative complications, being aware of the difference in comorbidities and extended hospitalization time for pediatric patients from URM groups undergoing open treatment for femoral shaft fractures can help improve overall patient well-being and healthcare costs. Regarding extended hospital stay, Kosuge et al. emphasized that reducing pediatric hospital stay following operative treatment is beneficial in all sectors of patient lives, including social, educational, and psychological [29]. Racial disparities are known to influence a child’s biological functioning, family relationships, and the likelihood of engagement in risky or unhealthy behaviors [30]. By becoming more knowledgeable of how these disparities influence healthcare, we as a profession can contribute to reducing disparities in an effort to lower costs, improve outcomes, and provide high-quality care for all patients [29,30].

The results of this study should be viewed in the context of its strengths and limitations. One strength of this study is that the NSQIP database is a large, nationwide database which allows us to obtain a large and diverse sample size of patients who underwent open treatment for a femoral shaft fracture. However, there are several limitations to this study. First, the NSQIP database only allows for the evaluation of 30-day postoperative complications, limiting the scope of evaluating long-term complications. Additionally, this database does not provide data on patient functional outcomes or pain scores. Second, the URM cohort was not stratified by specific racial/ethnic groups, which impedes the ability to make conclusions based on individual racial/ethnic groups. Another limitation is that we did not stratify by type of open treatment that patients underwent. By analyzing all open operative techniques as a whole, conclusions about specific procedures cannot be made.

## Conclusions

In conclusion, our study found that pediatric patients from URM groups are more likely to have an extended length of hospital stay compared to white pediatric patients. Our study did not find any other significant differences in 30-day postoperative complications, operative time, or transfusion requirement between the two cohorts. Future studies should investigate potential underlying causes for an increased hospital stay in URM groups, such as postoperative pain management, socioeconomic factors, or postoperative rehabilitation needs. This study adds to our understanding of differences in healthcare experiences and the effect of racial disparities in fracture care for pediatric patients.
